# Tree-based, two-stage risk factor analysis for postoperative sepsis based on Sepsis-3 criteria in elderly patients: A retrospective cohort study

**DOI:** 10.3389/fpubh.2022.1006955

**Published:** 2022-09-26

**Authors:** Xiaorong Peng, Chaojin Chen, Jingjing Chen, Yanlin Wang, Duo Yang, Chuzhou Ma, Zifeng Liu, Shaoli Zhou, Ziqing Hei

**Affiliations:** ^1^Department of Anesthesiology, The Third Affiliated Hospital of Sun Yat-sen University, Guangzhou, China; ^2^Big Data and Artificial Intelligence Center, The Third Hospital of Sun Yat-sen University, Guangzhou, China; ^3^Department of Anesthesiology, Jieyang People's Hospital, Jieyang, China; ^4^Department of Anesthesiology, Shantou Central Hospital, Shantou, China

**Keywords:** sepsis, risk factors, elderly, tree-based analysis, classification and regression tree

## Abstract

**Background:**

Sepsis remains the leading cause of postoperative death in elderly patients and is defined as organ dysfunction with proven or suspected infection according to Sepsis-3 criteria. To better avoid potential non-linear associations between the risk factors, we firstly used a tree-based analytic methods to explore the putative risk factors of geriatric sepsis based on the criteria in the study.

**Methods:**

Data of 7,302 surgical patients aged ≥ 65 years at the Third Affiliated Hospital of Sun Yat-sen University from January 2015 to September 2020 were collected. An analytic method that combined tree-based analysis with the method of Mantel-Haenszel and logistic regression was adopted to assess the association between 17 putative risk factors and postoperative sepsis defined by the Sepsis-3 guideline by controlling 16 potential confounding factors.

**Results:**

Among the 16 potential covariates, six major confounders were statistically identified by the tree-based model, including cerebrovascular diseases, preoperative infusion of red blood cells, pneumonia, age ≥ 75, malignant tumor and diabetes. Our analysis indicated that emergency surgery increases the risk of postoperative sepsis in elderly patients by more than six times. The type of surgery is also a crucial risk factor for sepsis, particularly transplantation and neurosurgery. Other risk factors were duration of surgery > 120 min, administration of steroids, hypoalbuminemia, elevated creatinine, blood urea nitrogen, hematocrit, platelets, glucose, white blood cell count, abnormal neutrophil-to-lymphocyte ratio and elevated hsCRP-to-albumin ratio.

**Conclusions:**

Our study uses an effective method to explore some risk factors for postoperative sepsis in elderly by adjusting many potential confounders and it can provide information for intervention design.

## Introduction

Sepsis is defined as organ dysfunction in patients with proven or suspected infection (1) and about 30% occurs after surgery (2). Sepsis remains the leading cause of postoperative mortality in surgical patients (3), particularly the elderly patients that are more susceptible to sepsis and septic shock (4, 5). The incidence of sepsis has been reported to be disproportionately higher in the elderly, and both short- and long-term survival and prognosis in older adult with postoperative sepsis were significantly worse compared with that in the young population (6, 7). As the population ages, age of patients undergoing surgery has been increasing, severe sepsis has been termed “a quintessential disease of aged” (8). Thus, geriatric sepsis after surgery represents a substantial health care burden worldwide and early prediction and intervention help to improve the outcomes of geriatric postoperative sepsis (9).

Previous definitions of sepsis were based largely on systemic inflammatory response syndrome (SIRS) and resulted in about 12.5% missed diagnosis (10) as well as misdiagnosis due to high sensitivity. The newly proposed Sepsis-3 criteria emphasize the importance of organ dysfunction determined by Sequential Organ Failure Assessment (SOFA) scores (1) and prognosis, and recognized in most countries. Limited studies have been carried out in the elderly patients and identified potential risk factors for geriatric postoperative sepsis on previous definitions of sepsis, such as age, comorbidities, and dependent function (11, 12). However, such studies were limited by previous definitions of sepsis. Moreover, earlier studies used the univariate analysis or logistic regression analysis with a hypothesis of linear associations between the risk factors and sepsis. However, multiple known and suspected confounding risk factors (such as age and comorbidities) must be considered during the analysis and potentially non-linear associations or complicated relationships between the potential risk factors and sepsis would exaggerate or conceal their underlying effects.

Machine learning is a novel method to address the complicated and non-linear interactions and tree-based model analysis is widely recognized as it takes into account the predictive value of all factors sequentially in a hierarchy of importance (13). Moreover, tree-based model typically produces a simple and easily interpreted decision tree, which can also stratify the population according to some confounding factors. Nevertheless, application of the tree-based model to analyze risk factors for postoperative sepsis in elderly patients has not been validated so far. Thus, tree-based analysis was used in this study to address effect of the potential confounding factors and further explore the putative risk factors of geriatric sepsis based on the Sepsis-3 criteria, which enable early prediction and intervention to improve the outcomes of geriatric surgery patients.

## Materials and methods

### Study design and patient selection

This retrospective cohort study was approved by the Research Ethics Committee at the Third Affiliated Hospital of Sun Yat-sen University, Guangzhou, China (No. [2019]02-609-02). All the relevant data were retrieved from the electronic health record system, a database created by extracting medical records from the hospital information system, laboratory information system, picture archiving and communication system, and Docare Anesthesia System. Consecutive elderly patients (aged ≥ 65 yrs) (14) undergoing surgery between January 2015 and September 2020 were eligible for inclusion. Patients who received general or regional anesthesia without intubation, had incomplete anesthetic and postoperative data were excluded.

### Data collection

#### Putative risk factors

Based on previous literature and our clinical experience, we collected 17 putative risk factors for postoperative sepsis that were explored in other surgical population, including the preoperative laboratory variables [white blood cell counts (WBC), hematocrit (HCT), red blood cell distribution width (RDW), platelets (PLT), blood glucose (GLU), albumin (ALB), blood urea nitrogen (BUN), serum creatinine (SCr), hypersensitive C-reactive protein (hsCRP), hypersensitive C-reactive protein-to-albumin ratio (CAR), neutrophil-to-lymphocyte ratio (NLR)], and intraoperative variables (timing of surgery, types of surgery, duration of surgery, administration of dexmedetomidine, ulinastatin and steroids). Among them, preoperative laboratory variables were divided into groups according to clinical significance, normal reference of results in our hospital, or previous studies (see for details in Supplementary Table 1).

#### Potential confounding factors

Meanwhile, we also enrolled 16 potential confounding factors that have been studied in the literature to confirm the association between putative risk factors and postoperative sepsis (11, 15–17), including demographic characteristics [age (65–74 or ≥ 75 years), sex, body mass index (< 18.5, 18.5–24 or ≥ 24 kg/m^2^), smoking and drinking], preoperative comorbidities [diabetes, coronary disease, cerebrovascular diseases, malignant tumor, pneumonia, hepatic failure, acute renal failure and numbers of preoperative comorbidities (0, 1–2 or ≥ 3)], and preoperative management related to acquired immunosuppression or immune dysregulation [chemical therapy, dialysis and infusion of red blood cells (RBC)].

#### Outcome definition

In this current study, sepsis was defined as life-threatening organ dysfunction caused by a dysregulated host response to infection and the diagnosis of sepsis was a SOFA score increase of ≥ 2 based on the Sepsis-3 criteria in the presence of proven or suspected infection (1). Infections were confirmed by microbiological cultures (excluding samples that are contaminated or thought to indicate colonialization) were classified as suspected if antibiotics were administered in addition to those used as standard prophylaxis, or if the surgeons documented a suspected infection in the EPR's clinical notes. Daily SOFA scores were calculated after surgery until discharge. Patients were divided into sepsis group and non-sepsis group based on the sepsis criteria^1^.

### Statistical analysis

As mentioned above, all continuous variables were divided into groups for the explainability in the clinical work (Table 1). Before grouping, we used means and modes to fill in missing values for continuous covariates and categorical covariates, respectively. All data were expressed as numbers (percentage) as all the continuous data were converted into categorical variables after grouping. Pearson's chi-square test was applied to compared categorical data.

**Table 1:** Clinical characteristics of the patients with or without postoperative sepsis.

**Characteristic**	**No. (%)**	**Non-sepsis**	**Sepsis**	***P*-value**
	***n* = 7,302**	***n* = 6,905**	***n* = 397**	
**Demographic factors**				
**Age**				< 0.001
65–74	5,368 (73.5%)	5,122 (74.2%)	246 (62.0%)	
≥75	1,934 (26.5%)	1,783 (25.8%)	151 (38.0%)	
**Sex**				0.002
Male	4,162 (57.0%)	3,905 (56.6%)	257 (64.7%)	
Female	3,140 (43.0%)	3,000 (43.4%)	140 (35.3%)	
**BMI (kg/m** ^**2**^ **)**				0.162
< 18.5	382 (5.23%)	353 (5.11%)	29 (7.30%)	
18.5–24	5,754 (78.8%)	5,448 (78.9%)	306 (77.1%)	
≥24	1,166 (16.0%)	1,104 (16.0%)	62 (15.6%)	
**Smoking**				0.271
No	6,496 (89.0%)	6,150 (89.1%)	346 (87.2%)	
Yes	806 (11.0%)	755 (10.9%)	51 (12.8%)	
**Drinking**				0.014
No	6,904 (94.5%)	6,540 (94.7%)	364 (91.7%)	
Yes	398 (5.45%)	365 (5.29%)	33 (8.31%)	
**Preoperative Comorbidities**				
**Diabetes**				< 0.001
No	4,776 (65.4%)	4,562 (66.1%)	214 (53.9%)	
Yes	2,526 (34.6%)	2,343 (33.9%)	183 (46.1%)	
**Coronary disease**				0.046
No	6,825 (93.5%)	6,464 (93.6%)	361 (90.9%)	
Yes	477 (6.53%)	441 (6.39%)	36 (9.07%)	
**Cerebrovascular diseases**				< 0.001
No	7,190 (98.5%)	6,827 (98.9%)	363 (91.4%)	
Yes	112 (1.53%)	78 (1.13%)	34 (8.56%)	
**Malignant tumor**				< 0.001
No	4,616 (63.2%)	4,399 (63.7%)	217 (54.7%)	
Yes	2,686 (36.8%)	2,506 (36.3%)	180 (45.3%)	
**Hepatic failure**				< 0.001
No	7,225 (98.9%)	6,842 (99.1%)	383 (96.5%)	
Yes	77 (1.05%)	63 (0.91%)	14 (3.53%)	
**Pneumonia**				< 0.001
No	6,838 (93.6%)	6,500 (94.1%)	338 (85.1%)	
Yes	464 (6.35%)	405 (5.87%)	59 (14.9%)	
**Acute kidney injury**				0.048
No	7,167 (98.2%)	6,783 (98.2%)	384 (96.7%)	
Yes	135 (1.85%)	122 (1.77%)	13 (3.27%)	
**Number of preoperative comorbidities**				< 0.001
0	2,684 (36.8%)	2,596 (37.6%)	88 (22.2%)	
1–2	4,352 (59.6%)	4,080 (59.1%)	272 (68.5%)	
≥3	266 (3.64%)	229 (3.32%)	37 (9.32%)	
**Preoperative management**				
**Chemical therapy**				0.02
No	7,262 (99.5%)	6,871 (99.5%)	391 (98.5%)	
Yes	40 (0.55%)	34 (0.49%)	6 (1.51%)	
**Dialysis**				0.001
No	6,920 (94.8%)	6,558 (95.0%)	362 (91.2%)	
Yes	382 (5.23%)	347 (5.03%)	35 (8.82%)	
**Infusion of RBC**				< 0.001
No	7,123 (97.5%)	6,756 (97.8%)	367 (92.4%)	
Yes	179 (2.45%)	149 (2.16%)	30 (7.56%)	
**Intraoperative variables**				
**Timing of surgery**				< 0.001
Elective	6,774 (92.8%)	6,510 (94.3%)	264 (66.5%)	
Emergency	528 (7.23%)	395 (5.72%)	133 (33.5%)	
**Type of surgery**				< 0.001
Abdominal and urogenital surgery	4,216 (57.7%)	3,999 (57.9%)	217 (54.7%)	
Cardiovascular and thoracic surgery	591 (8.09%)	548 (7.94%)	43 (10.8%)	
Neurosurgery	445 (6.09%)	363 (5.26%)	82 (20.7%)	
Orthopedic surgery	1,069 (14.6%)	1,042 (15.1%)	27 (6.80%)	
Transplantation	86 (1.18%)	64 (0.93%)	22 (5.54%)	
Head and neck surgery	895 (12.3%)	889 (12.9%)	6 (1.51%)	
**Duration of surgery (min)**				< 0.001
≤ 120	3,169 (43.4%)	3,084 (44.7%)	85 (21.4%)	
>120	4,133 (56.6%)	3,821 (55.3%)	312 (78.6%)	
**Administration of dexmedetomidine**				0.755
No	4,074 (55.8%)	3,849 (55.7%)	225 (56.7%)	
Yes	3,228 (44.2%)	3,056 (44.3%)	172 (43.3%)	
**Administration of ulinastatin**				< 0.001
No	6,086 (83.3%)	5,839 (84.6%)	247 (62.2%)	
Yes	1,216 (16.7%)	1,066 (15.4%)	150 (37.8%)	
**Administration of steroids**				< 0.001
No	5,011 (68.6%)	4,809 (69.6%)	202 (50.9%)	
Yes	2,291 (31.4%)	2,096 (30.4%)	195 (49.1%)	
**Preoperative laboratory variables**				
**WBC**				< 0.001
Normal	6,073 (83.2%)	5,803 (84.0%)	270 (68.0%)	
Abnormal	1,229 (16.8%)	1,102 (16.0%)	127 (32.0%)	
**HCT**				< 0.001
Normal	2,585 (35.4%)	2,493 (36.1%)	92 (23.2%)	
Abnormal	4,717 (64.6%)	4,412 (63.9%)	305 (76.8%)	
**RDW**				< 0.001
≤ 0.15	6,386 (87.5%)	6,075 (88.0%)	311 (78.3%)	
>0.15	916 (12.5%)	830 (12.0%)	86 (21.7%)	
**PLT**				0.001
Normal	6,501 (89.0%)	6,169 (89.3%)	332 (83.6%)	
Abnormal	801 (11.0%)	736 (10.7%)	65 (16.4%)	
**ALB (g/L)**				< 0.001
≥36	1,435 (19.7%)	1,271 (18.4%)	164 (41.3%)	
< 36	5,867 (80.3%)	5,634 (81.6%)	233 (58.7%)	
**BUN (mmol/L)**				< 0.001
≤ 8.2	6,341 (86.8%)	6,044 (87.5%)	297 (74.8%)	
>8.2	961 (13.2%)	861 (12.5%)	100 (25.2%)	
**SCr (μmol/L)**				< 0.001
≤ 116	6,649 (91.1%)	6,335 (91.7%)	314 (79.1%)	
>116	653 (8.94%)	570 (8.25%)	83 (20.9%)	
**GLU (mmol/L)**				< 0.001
≤ 10	6,855 (93.9%)	6,509 (94.3%)	346 (87.2%)	
>10	447 (6.12%)	396 (5.73%)	51 (12.8%)	
**hsCRP (mg/L)**				0.542
≤ 3	178 (2.44%)	166 (2.40%)	12 (3.02%)	
>3	7,124 (97.6%)	6,739 (97.6%)	385 (97.0%)	
**CAR**				< 0.001
< 0.278	6,486 (88.8%)	6,201 (89.8%)	285 (71.8%)	
≥0.278	816 (11.2%)	704 (10.2%)	112 (28.2%)	
**NLR**				< 0.001
≤ 3.75	5,405 (75.2%)	5,188 (76.4%)	217 (54.7%)	
>3.75	1,787 (24.8%)	1,607 (23.6%)	180 (45.3%)	

BMI, body mass index; RBC, red blood cell; WBC, white blood cell count; HCT, hematocrit; RDW, red blood cell distribution width; PLT, platelet; ALB, albumin; BUN, blood urea nitrogen; SCr, serum creatinine; GLU, blood glucose; hsCRP, hypersensitive C-reactive protein; CAR, hsCRP-to-albumin ratio; NLR, neutrophil-to-lymphocyte ratio.

To better find the effects of confounding factors on the incidence of postoperative sepsis, we applied a tree-based model (18, 19), also known as a classification and regression tree, to stratify the study population into relatively homogeneous subgroups based on a subset of the 16 confounding factors selected in the study. Tree-based model considers the divide and conquer method of decision tree induction. The algorithm identifies the most important independent variable and sets it as the root node, before forking to the next best variable. The algorithm works in a top-down fashion, from the root node to the internal nodes and finally to the terminal leaf nodes. In this study, we found the risk factors that best divided the sample into two groups at first. The best was defined as the partition that maximizes the sum of squares between groups (or, equivalently, minimizes the sum of squares of errors within groups). This process is applied recursively to each subgroup separately until the subgroup reaches the minimum sample (set to 20 in our analysis, the default value in part) or until the model fit cannot be improved. As the generated model is often too complex and may over-fit the data, the second stage of the process involves using cross-validation to prune the complete tree. We used stratified 10-fold cross-validation to assess model fit across a range of model complexity and select the optimal (pruned) tree by examining cross-validation errors in relation to model complexity. We followed the default parameter selection and finally chose the highest point of test scores in red to build the tree (see Supplementary Figure 1). In this study, three tree models were derived from different confounding factors. Model 1 based on demographic factors, model 2 based on demographic factors and preoperative comorbidities, and model 3 based on all the potential confounding factors were developed. Akaike information criterion (AIC) that focus on model accuracy and complexity was adopted for model selection.

To further control for confounders, we used the Mantel-Haenszel method (20, 21) to adjust for the effect of 17 putative risk factors on postoperative sepsis and derived adjusted relative risks (RRs). Similarly, we explored the marginal association between sepsis and 17 potential risk factors using a univariate logistic analysis. In addition, we explored the relationship between confounding factors and postoperative sepsis through following tree model screening.

For sensitivity analysis, logistic regression (LR) was performed and the adjusted odd ratios (ORs) were calculated to explore the association between the 17 potential risk factors and sepsis. Notably, the significant confounding factors were also taken into consideration.

All *P*-values were two sided, and *P* < 0.05 was considered as statistically significant. The construction and pruning of the tree model were done in Python 3.8, and the other statistical analyses were performed using R version 3.6.2 software (Vienna, Austria).

## Results

### Population characteristics

**Figure 1:**
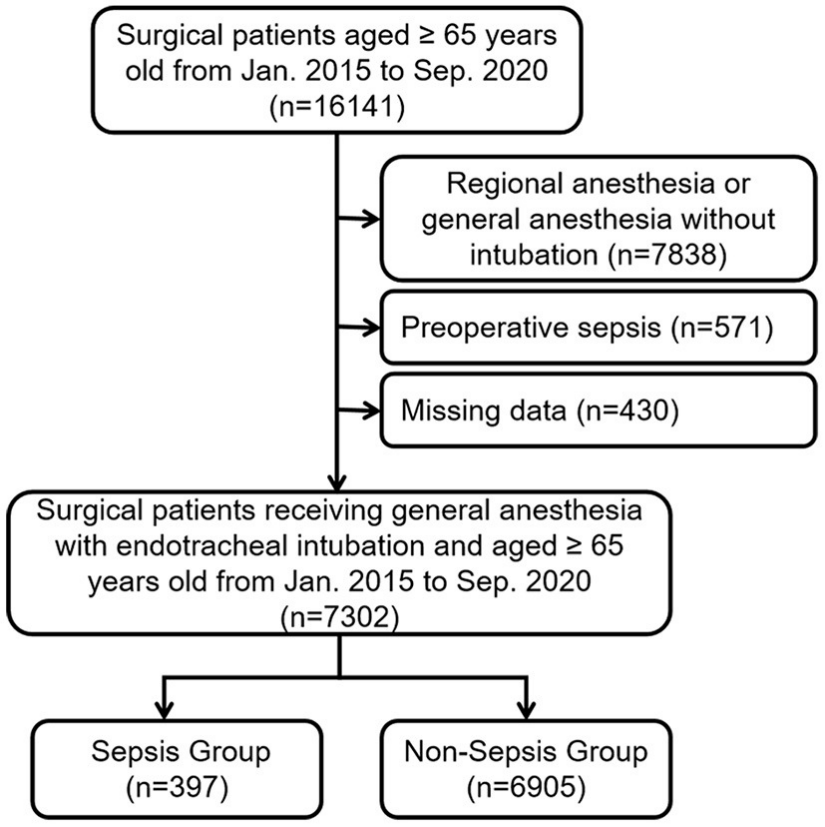
STROBE flowchart of patient enrollment in this study.

A total of 16,141 patients aged ≥ 65 yrs were collected, of whom 8,839 were excluded because they did not meet the inclusion criteria, leaving 7,302 to be enrolled in the final analysis (Figure 1). The mean age was 70 yrs, with approximately three quarters of patients falling into the 65–74 yrs group. The majority of patients were male (4,162, 57.0%). Three hundred and ninety-seven (5.4%) patients developed postoperative sepsis. The clinical characteristics of the patients were presented in Table 1, including 17 putative risk factors and 16 covariates.

### Analysis of marginal association based on univariate logistic analysis

The marginal association between sepsis and the 17 putative risk factors were shown in Table 2. We found that 15 of 17 variables were significantly associated with sepsis. Emergency surgery showed a significant marginal correlation as 25.19% of patients who underwent emergency surgery developed sepsis compared to 3.90% of patients who underwent elective surgery. The relative risk of sepsis in patients undergoing emergency surgery was 8.30 (95% CI 6.57–10.45) compared to the group undergoing elective surgery. Patients undergoing neurosurgery (RR 4.16, 95% CI 3.12–5.52) or transplantation (RR 6.33, 95% CI 3.64–10.65) had a higher risk of postoperative sepsis compared to those undergoing abdominal and genitourinary surgery; however, patients undergoing orthopedic surgery (RR 0.48, 95% CI 0.31–0.72) or head and neck surgery (RR 0.12, 95% CI 0.05–0.28) had a lower risk. Duration of surgery > 120 min (RR 2.96, 95% CI 2.33–3.80) and preoperative hypoalbuminemia (RR 3.12, 95% CI, 2.53–3.84) were associated with a higher risk of sepsis. Patients with elevated CAR or NLR were at higher risk of suffering postoperative sepsis (RR 3.46, 95% CI, 2.74–4.35 and RR 2.67, 95% CI 2.16–3.29, respectively). Meanwhile, intraoperative administration of steroids, ulinastatin, preoperative abnormal WBC, HCT, and PLT, elevated RDW, BUN, SCr, and GLU were also associated with postoperative sepsis.

**Table 2:** Putative risk factors for sepsis.

**Characteristic**	**Crude**		**Model 1**		**Model 2**		**Model 3**		**LR**	
	**RR^a^**	**95%CI**	**RR^b^**	**95%CI**	**RR^c^**	**95%CI**	**RR^d^**	**95%CI**	**OR^e^**	**95%CI**
**Timing of surgery**										
Elective	Ref.		Ref.		Ref.		Ref.		Ref.	
Emergency	8.30	(6.57, 10.45)	7.84	(6.21, 9.91)	6.54	(5.11, 8.38)	6.66	(5.13, 8.64)	7.54	(5.79, 9.79)
**Type of surgery**										
Abdominal and urogenital surgery	Ref.		Ref.		Ref.		Ref.		Ref.	
Cardiovascular and thoracic surgery	1.45	(1.00,2.04)	1.47	(1.05, 2.07)	1.36	(0.96, 1.92)	1.35	(0.95, 1.92)	1.38	(0.97, 1.94)
Neurosurgery	4.16	(3.12,5.52)	4.31	(3.26, 5.70)	3.32	(2.40, 4.61)	3.85	(2.73, 5.42)	4.20	(3.00, 5.82)
Orthopedic surgery	0.48	(0.31,0.72)	0.40	(0.26, 0.61)	0.40	(0.27, 0.61)	0.47	(0.31, 0.72)	0.51	(0.32, 0.76)
Transplantation	6.33	(3.64, 10.65)	5.66	(3.42, 9.38)	5.25	(3.15, 8.75)	4.27	(2.44, 7.47)	4.18	(2.38, 7.08)
Head and neck surgery	0.12	(0.05, 0.28)	0.14	(0.06, 0.31)	0.14	(0.06, 0.32)	0.16	(0.07, 0.36)	0.16	(0.06, 0.33)
**Duration of surgery (min)**										
≤ 120	Ref.		Ref.		Ref.		Ref.		Ref.	
>120	2.96	(2.33, 3.80)	3.05	(2.39, 3.90)	3.17	(2.47, 4.08)	2.89	(2.25, 3.72)	2.95	(2.29, 3.82)
**Administration of dexmedetomidine**										
No	Ref.		Ref.		Ref.		Ref.		Ref.	
Yes	0.96	(0.78, 1.18)	1.04	(0.84, 1.27)	1.08	(0.87, 1.33)	1.07	(0.87, 1.32)	1.06	(0.86, 1.31)
**Administration of ulinastatin**										
No	Ref.		Ref.		Ref.		Ref.		Ref.	
Yes	3.33	(2.68, 4.11)	3.18	(2.57, 3.94)	3.05	(2.45, 3.79)	2.83	(2.27, 3.54)	2.83	(2.26, 3.53)
**Administration of steroids**										
No	Ref.		Ref.		Ref.		Ref.		Ref.	
Yes	2.21	(1.81, 2.71)	2.22	(1.81, 2.72)	2.23	(1.81, 2.75)	2.23	(1.81, 2.74)	2.24	(1.81, 2.76)
**WBC**										
Normal^#^	Ref.		Ref.		Ref.		Ref.		Ref.	
Abnormal^##^	2.48	(1.98, 3.08)	2.35	(1.88, 2.93)	1.9	(1.50, 2.40)	1.86	(1.47, 2.35)	1.92	(1.51, 2.43)
**HCT**										
Normal^#^	Ref.		Ref.		Ref.		Ref.		Ref.	
Abnormal^##^	1.87	(1.48, 2.39)	1.77	(1.39, 2.24)	1.6	(1.25, 2.03)	1.51	(1.18, 1.93)	1.53	(1.20, 1.96)
**RDW**										
≤ 0.15	Ref.		Ref.		Ref.		Ref.		Ref.	
>0.15	2.02	(1.57, 2.59)	1.98	(1.54, 2.54)	1.87	(1.45, 2.42)	1.52	(1.16, 2.00)	1.49	(1.12, 1.97)
**PLT**										
Normal^#^	Ref.		Ref.		Ref.		Ref.		Ref.	
Abnormal^##^	1.64	(1.23, 2.15)	1.65	(1.25, 2.18)	1.56	(1.18, 2.08)	1.32	(0.99, 1.76)	1.30	(0.96, 1.73)
**ALB (g/L)**										
≥36	Ref.		Ref.		Ref.		Ref.		Ref.	
< 36	3.12	(2.53, 3.84)	2.84	(2.30, 3.51)	2.50	(2.02, 3.10)	2.27	(1.83, 2.82)	2.43	(1.95, 3.03)
**BUN (mmol/L)**										
≤ 8.2	Ref.		Ref.		Ref.		Ref.		Ref.	
>8.2	2.36	(1.86, 2.99)	2.17	(1.7, 2.76)	2.09	(1.64, 2.68)	2.12	(1.66, 2.72)	2.14	(1.66, 2.74)
**SCr**										
≤ 116	Ref.		Ref.		Ref.		Ref.		Ref.	
>116	2.94	(2.26, 3.78)	2.74	(2.11, 3.56)	2.62	(2.01, 3.42)	2.59	(1.98, 3.38)	2.58	(1.96, 3.37)
**GLU (mmol/L)**										
≤ 10	Ref.		Ref.		Ref.		Ref.		Ref.	
>10	2.42	(1.76, 3.28)	2.38	(1.74, 3.25)	1.90	(1.38, 2.61)	1.96	(1.42, 2.7)	1.92	(1.37, 2.66)
**hsCRP (mg/L)**										
≤ 3	Ref.		Ref.		Ref.		Ref.		Ref.	
>3	0.79	(0.46, 1.51)	0.77	(0.42, 1.39)	0.89	(0.48, 1.64)	1.02	(0.55, 1.9)	1.00	(0.56, 1.94)
**CAR**										
< 0.278	Ref.		Ref.		Ref.		Ref.		Ref.	
≥0.278	3.46	(2.74, 4.35)	3.21	(2.54, 4.05)	2.56	(2.00, 3.27)	2.40	(1.88, 3.06)	2.67	(2.07, 3.42)
**NLR**										
≤ 3.75	Ref.		Ref.		Ref.		Ref.		Ref.	
>3.75	2.67	(2.17, 3.27)	2.48	(2.01, 3.05)	2.13	(1.73, 2.64)	2.09	(1.69, 2.59)	2.19	(1.76, 2.71)

^*a*^The crude relative risk is marginal association between sepsis and putative factors by using univariate logistic analysis.

^*b*^ The relative risk is estimated from the method of Mantel-Haenszel based on the tree model 1 including only demographic factors.

^*c*^The relative risk is estimated from the method of Mantel-Haenszel based on the tree model 2 including demographic factors and preoperative comorbidities.

^*d*^The relative risk is estimated from the method of Mantel-Haenszel based on the tree model 3 including all the potential confounding factors.

^*e*^The odds ratio is obtained from logistic regression concerning the one-staged interaction of age and pneumonia.

^#^Normal WBC, HCT, PLT are referred to 3.5–9.5(10^9^/L), 0.4–0.5, and 100–350(10^9^/L), respectively.

^*##*^Abormal WBC, HCT, PLT are referred to the values beyond the normal range respectively.LR, logistic regression; RR, relative risk; OR, odds ratio; CI, confidence interval; WBC, white blood cell count; HCT, hematocrit; RDW, red blood cell distribution width; PLT, platelet; ALB, albumin; BUN, blood urea nitrogen; SCr, serum creatinine; GLU, blood glucose; hsCRP, hypersensitive C-reactive protein; CAR, hsCRP-to-albumin ratio; NLR, neutrophil-to-lymphocyte ratio.

### Construction of tree models

**Figure 2:**
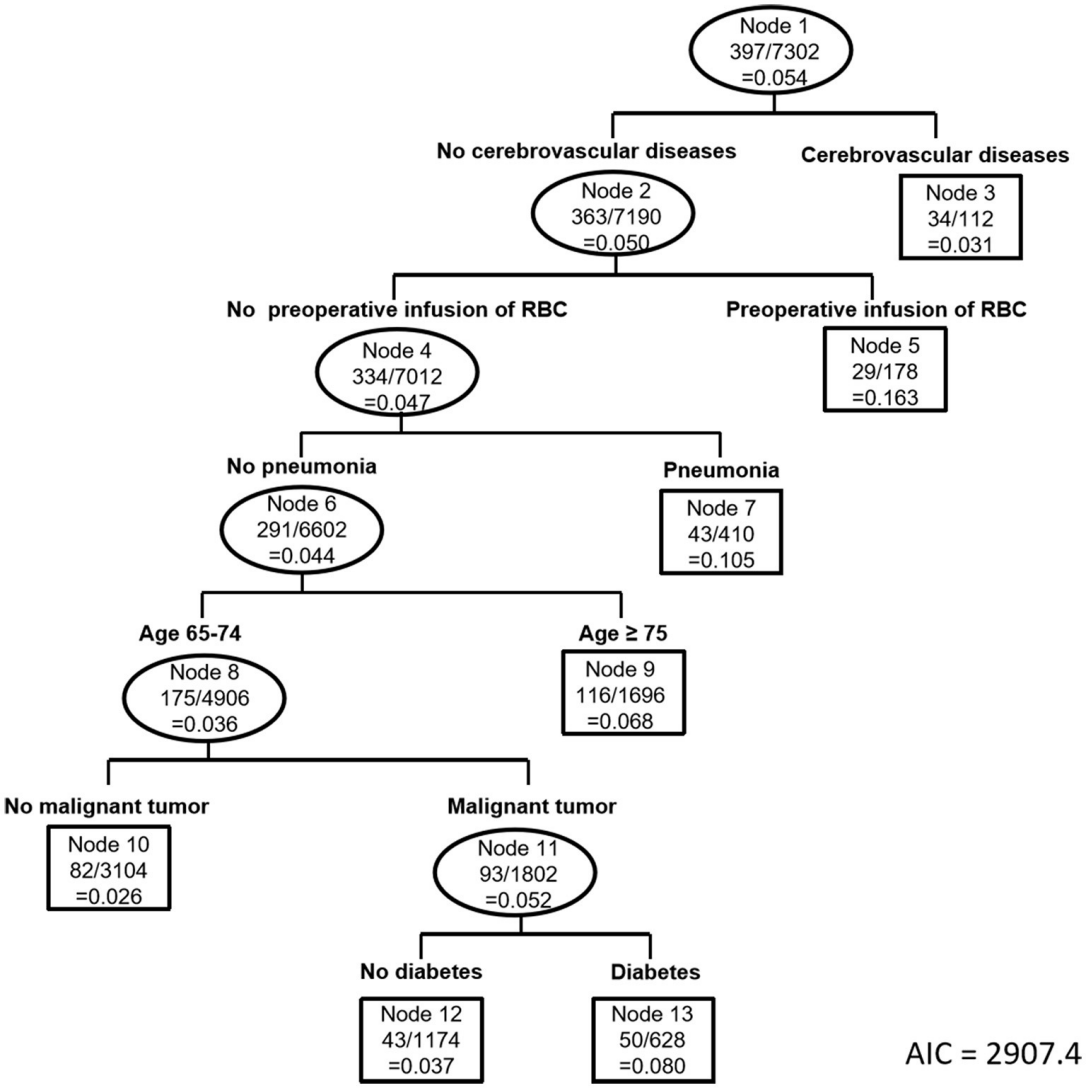
Tree structure derived from all the potential confounding factors (Model 3).

**Figure 3:**
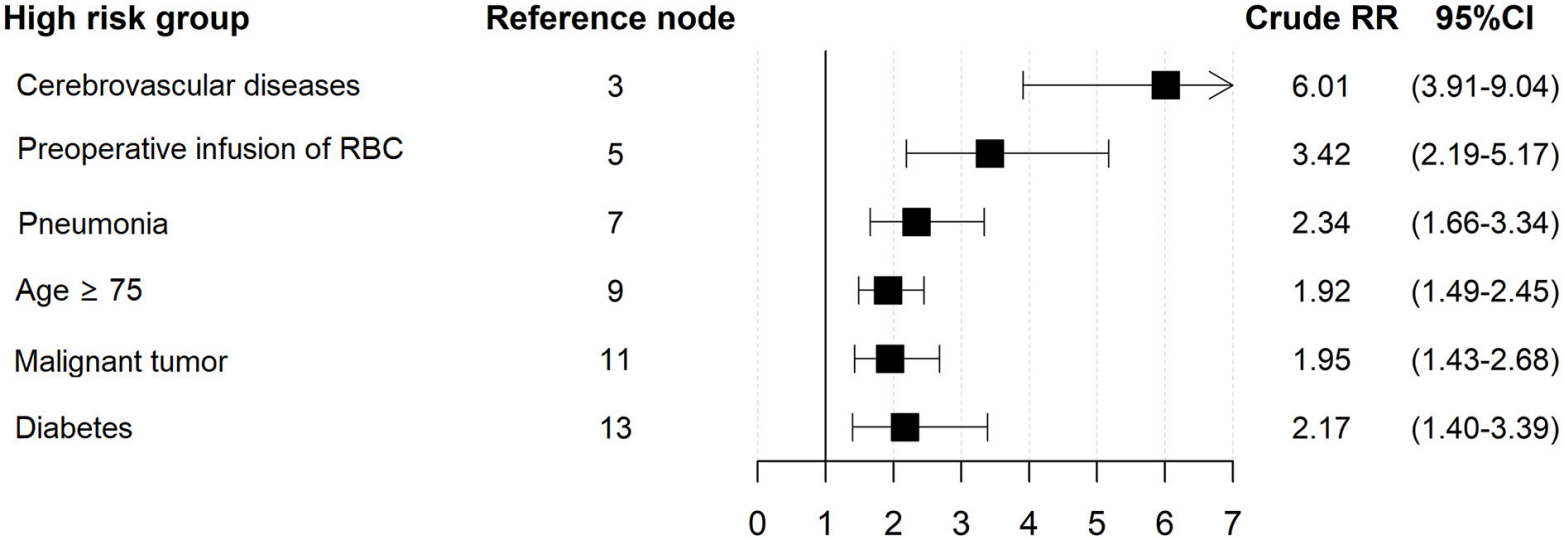
Crude relative risks for pairwise nodes of the tree model.

The goodness of fit of the 3 tree models was ranked according to AIC, with the one having the lowest AIC being the best. Model 1 showed an AIC of 3056.8, model 2 showed an AIC of 2943.7, and model 3 showed the lowest AIC of 2907.4. Therefore, model 3 derived from all the potential confounders is the best tree model of postoperative sepsis in the elderly and detailed in Figure 2. Among the 16 potential covariates, six main confounding factors were statistically identified by the tree model, including cerebrovascular diseases, preoperative infusion of RBC, pneumonia, age ≥ 75 yrs, malignant tumor and diabetes. We used these six variables for classification and generated seven subgroups bases on the magnitude of risk. Each subgroup was designated by a probabilistic combination of potential confounders and the incidence of postoperative sepsis. Specifically, the total population was first divided into two groups based on the presence or absence of preoperative cerebrovascular diseases. Notably, the incidence of sepsis was 30.3% and 5.0% in patients with and without cerebrovascular diseases, respectively [RR 6.01, 95%CI (3.91–9.04), Figure 3]. The pattern of risk observed in each subgroup revealed interactions between a set of potential confounding variables. For example, we found that patients without cerebrovascular diseases, preoperative infusion of RBC or pneumonia, the risk of development of postoperative sepsis depended on the age. Patients aged ≥ 75 yrs had a higher risk of postoperative sepsis compared to patients aged 65–74 yrs [RR 1.92, 95% CI (1.49–2.45), Figure 3]. There was also an association between diabetes, malignant tumors and age, reflected in the fact that comorbid diabetes increases the risk of postoperative sepsis in patients aged 65–74 yrs with malignant tumors but not cerebrovascular diseases, preoperative transfusion of red blood cells or pneumonia. The model showed that among the 16 potential confounders identified, the presence of cerebrovascular disease was the most important risk factor for the incidence of sepsis. However, in patients without cerebrovascular disease, the risk was dependent on age and other preoperative conditions. It is worth mentioning that interactions between variables may be found in specific subgroup.

Besides, model 1 indicated that age ≥ 75 yrs and drinking were confounding factors for postoperative sepsis in the elderly patients if only demographic factors were taken into account. Model 2 showed that cerebrovascular diseases, pneumonia, age ≥ 75 yrs, and diabetes were confounding factors for postoperative sepsis if both demographic factors and comorbidities were considered (Supplementary Figure 2).

### Adjusted risks based on tree models and LR

To further control for the six stratified confounders shown in model 3, we then applied the Mantel-Haenszel method in the stratified sample to derive adjusted relative risks for 17 hypothetical risk factors. We found that 14 of the factors had a significant effect. Patients undergoing emergency surgery had a higher risk of sepsis after surgery under general anesthesia (RR 6.66, 95% CI 5.13–8.64); We found that the risk of postoperative sepsis for neurosurgery (RR 3.85, 95% CI 2.73–5.42) and transplantation (RR 4.27, 95% CI 2.44–7.47) surgery were higher; however, the risk was lower for orthopedic surgery (RR 0.47, 95% CI 0.31–0.72) and head and neck surgery (RR 0.16, 95% CI 0.07–0.36) compared to abdominal and urogenital surgery. In addition, patients with hypoalbuminemia, duration of surgery > 120 min, abnormal WBC, and elevated NLR, CAR, creatinine also suffered a greater risk of postoperative sepsis (Table 2). Additionally, the relative risks estimated from the method of Mantel-Haenszel based on the model 1, model 2, and model 3 gave very close answers except PLT.

For sensitivity analysis, we also applied logistic regression to obtain the adjusted odds ratio of 17 hypothetical risk factors, as shown in the last two columns in Table 2. Notably, we found a first-order interaction between pneumonia and age (*P* = 0.0418), so we considered the first-order interactions between these two variables as well as the main effects of the other four confounding factors. After performing the positive and stepwise regression analysis among the 17 potential risk factors, we found the adjusted ORs were consistent with the adjusted RRs of the risk factors. Therefore, for this study, it seems unwarranted to adjust the risks for the 17 potential risk factors. However, this does not mean that it is not necessary to calculate the adjusted RR because we cannot draw this conclusion without them.

## Discussion

Early prediction and rapid treatment are crucial to improving outcomes of patients with postoperative sepsis (22), especially in the elderly patients that are more susceptible to sepsis and associated with higher mortality (23). Early studies have recognized many risk factors for sepsis, but few of them are carried out in elderly patients based on the Sepsis-3 Criteria and most of them assumed a linear relationship between risk factors and sepsis. This report has utilized a tree-based method to explore 17 assumed putative risk factors for geriatric postoperative sepsis by adjusting 16 potential confounding factors, considering the intricate interactions among variables. Accordingly, our study has demonstrated that several modifiable and unmodifiable risk factors are related to postoperative sepsis. Risk factors, such as emergency surgery (6.7 times), longer duration of surgery (2.9 times), Steroids (2.2 times), preoperative higher CAR (2.4 times), higher NLR (2.1 times) hypoalbuminemia (2.3 times) and elevated SCr (2.6 times) increased the risk of postoperative sepsis in the elderly patients significantly.

In the current study, three tree models were constructed for control of confounding factors for postoperative sepsis based on including different variables. In the tree-based analysis, the model 3 included all the covariates while the other models only included partial confounding factors. Model 3 was optimal, with the minimum AIC of 2907.4. As showed in Figure 3, the elderly patients with cerebrovascular diseases were at higher risk of developing sepsis than those without sepsis, which is previously reported in some studies based on Sepsis-1 criteria (24, 25). However, the role of cerebrovascular diseases in sepsis remains uncertain (26). Our study gives positive support to this argument. Specifically, with or without cerebrovascular diseases was chosen as the first splitter variable in our model, which suggests the strongest significance in risk for postoperative sepsis for the aged surgical patients. Transfusion may significantly increase risk of infection by down-regulating of immunologic functions or bacterial contamination of blood components (7, 27), and has been reported to be a risk factor for postoperative sepsis. Interestingly, our study showed that preoperative blood transfusion was a risk factor only for those without cerebrovascular diseases. Besides, age ≥ 75 yrs, pneumonia, diabetes and malignant tumor were also found to be associated with postoperative sepsis in elderly patients in the study.

Compared with the multivariable logistic regression analysis, non-parametric tree-based methods allow for the underlying complicated relationships between the risk factors. Tree-based methods also select the variables in order of importance and subgroup the population based on the risk. Since a large number of predictors are considered stratification factors/variables (particularly categorical ones), tree-based model is an appropriate alternative method, in view of laborious work in logistic regression model. In this study, we aim to address specific hypotheses by estimating adjusted odds ratio and relative risks for postoperative sepsis in the surgical elderly patients. Therefore, we do not conduct a pure tree-based model based on both the potential risk factors and confounding factors to find out the risk population. Instead, we used both logistic regression and the Mantel-Haenszel method after the tree-based analysis for assessment to examine the effects of risk factors (presented as RRs or ORs).

Our study also reported several strong factors associated with postoperative sepsis in the elderly surgical patients, including emergency surgery, duration of surgery, type of surgery and intraoperative administration of steroids, which are consistent with previous reported risk factors (15, 16, 28–32). Notably, we found that emergency surgery increased the risk of postoperative sepsis over 6-fold after regarding the confounders, and this was greater than other investigations that reported twice (29) or three-time risk (15) of emergency surgery contributing to postoperative sepsis in adults. The results revealed that in aged patients undergoing emergency surgery, more early intervention should be adopted purposefully to prevent postoperative sepsis.

Besides the emergency surgery, multiple preoperative laboratory results had been reported to predict sepsis but the causative interactions remain confusing (33). Meanwhile, most of risk factors of sepsis are not well defined and focus on the susceptibility to infection (34, 35). In the study, we applied the Mantel-Haenszel method in the stratified sample to derive adjusted relative risks and odds ratios for 17 hypothetical risk factors and found that preoperative abnormal WBC and HCT, elevated RDW, BUN, SCr and GLU were significantly associated with postoperative sepsis. All the variables were explainable according to our clinical experience. For instance, decreased renal function (elevated BUN and SCr) contributes to immunocompromised status through retention of uremic toxins and malnutrition, hence, increases development of infection and sepsis (36, 37). We also found that poor GLU control (preoperative GLU > 10 mmol/L) (38) was a risk factor of sepsis and this might be attributed to the effect of glycemia on altering the immune system through decrease of secretion of inflammatory cytokines, depression in neutrophils and T cells function, resulting in the risk for infection and delaying the recovery from infection (39). The results in our study also confirmed the association between sepsis and inflammatory biomarkers, including increased WBC, RDW (40), NLR (41, 42) and CAR (43). Notably, there exists little difference among the adjusted RRs obtained by Mantel-Haenszel method based on different tree models and adjusted ORs obtained by logistic regression, indicating that our results were stable and reliable to predict postoperative sepsis in the elderly.

Consistent with earlier study (44), our results showed hypoalbuminemia an indicator of malnutrition and inflammation that increases the risk for infection and sepsis, but no significant correlation between increased hsCRP and postoperative sepsis was found. Instead, elevated CAR was shown to indicate higher risk of sepsis, compared with hypoalbuminemia and the other inflammatory biomarkers. As we earlier reported, we think this might be attributed to the low specificity of hsCRP and the CAR is thought to integrate information provided by hsCRP and albumin, and higher CAR is often associated with nutritional deficiency and severe inflammation, which makes it superior to other systemic inflammation biomarkers.

In this study, we revealed several factors that need to be addressed and modified to decrease the risk for developing postoperative sepsis. For examples, decrease of unnecessary blood transfusion, correcting hypoalbuminemia, well control of preoperative blood glucose and infection status, and shortening the operation time may decrease the risk of postoperative sepsis. Although we found the intraoperative administration of ulinastatin was associated with postoperative sepsis, which is inconsistent with its anti-inflammatory effect (45), we think this might be attributed to the fact that patients received intra-operative ulinastatin might have worse perioperative conditions and received a higher grade of surgery.

Our study may have several limitations. Firstly, the risk factor analysis was developed in a single-center study, and future multi-center study will be needed for generalization and validation. Secondly, the study was performed retrospectively, for which possible residual confounding may occur and we couldn't explore the exact effect of ulinastatin administration on patients with the same condition. Thirdly, analysis was restricted to the variables available from the electronic health record system; hence, some potential factors such as bacterial species and infection site cannot be evaluated. Finally, the cut-off points of the preoperative laboratory test were grouped mostly according to the normal ranges in our hospital instead of the optimal cut-off point. We consider the little variation of results caused technique for testing in different hospitals and attention will be attracted by abnormal results.

## Conclusion

In conclusion, we developed a tree-based analysis for postoperative sepsis defined by the newly introduced Sepsis-3 criteria in elderly patients, taking non-linear interactions into consideration, and revealed many risk factors for postoperative sepsis among the surgical aged patients, including preoperative clinical laboratory results and surgery-related factors. It provides evidence for clinical decision-making by modification of some preoperative risk factors and early identification of high-risk surgical patients after surgery.

## Data availability statement

The raw data supporting the conclusions of this article will be made available by the authors, without undue reservation.

## Ethics statement

The studies involving human participants were reviewed and approved by the Ethnic Committee of The Third Affiliated Hospital of Sun Yat-sen University (No. [2019]02-609-02). Written informed consent for participation was not required for this study in accordance with the national legislation and the institutional requirements.

## Author contributions

ZL, SZ, and ZH designed this research. XP and CC collected and anonymized the original data. XP, CC, and JC designed and guided the process of data cleaning, model building, and statistics. CC, DY, CM, and YW defined the rules of data extraction and interpreted patient data. ZL and JC performed data cleaning and built the tree-based model. XP and CC equally contributed in writing the manuscript. DY, CC, and JC revised the manuscript. All authors read and approved the final manuscript.

## Funding

This study was supported by the National Natural Science Foundation of China (Grant Nos. 82102297, 82072218, and 81974296), and Natural Science Foundation of Guangdong Province (Grant Nos. 2018A0303130224, 2019A1515110020, 2020A1515010153, and 2022A1515012603), Science and Technology Program of Guangzhou, China (Grant No. 202002020047), Young Talent Support Project of Guangzhou Association for Science and Technology (Grant No. QT20220101257), and the Fundamental Research Funds for the Central Universities of China (Grant No. 22qntd3401).

## Conflict of interest

The authors declare that the research was conducted in the absence of any commercial or financial relationships that could be construed as a potential conflict of interest.

## Publisher's note

All claims expressed in this article are solely those of the authors and do not necessarily represent those of their affiliated organizations, or those of the publisher, the editors and the reviewers. Any product that may be evaluated in this article, or claim that may be made by its manufacturer, is not guaranteed or endorsed by the publisher.

## Footnotes

^1^For Original Research articles, please note that the Material and Methods section can be placed in any of the following ways: before Results, before Discussion or after Discussion.
