# Supplementary material for: Tree-based, two-stage risk factor analysis for postoperative sepsis based on Sepsis-3 criteria in elderly patients: A retrospective cohort study
Source: Front Public Health. 2022 Sep 26;10:1006955. doi: 10.3389/fpubh.2022.1006955 (PMC9550002; doi:10.3389/fpubh.2022.1006955)
Supplement: Supplementary file 1 [file Presentation_1.pdf]

Supplementary Material

1 Supplementary Data

Laboratory variables were grouped according to:

(1) Normal range（**Supplementary Table 1**） and clinical significance
①HCT, WBC, PLT were divided into normal group and abnormal group according to normal range of laboratory tests in our hospital.

②The upper normal limits of hsCRP, RDW, BUN and SCr were selected as cut-off points for grouping, respectively.

③The lower normal limit of ALB was selected as cut-off point.

④The cut-off point of NLR was obtained from the ratio of upper normal limits of percentage of neutrophils (0.75) to lower normal limits of percentage of lymphocytes (0.2).

(2) Literature

①According to the guideline of Perioperative Hyperglycemia Management, preoperative blood glucose > 10 mmol/L is recognized as poor glucose control.

②CAR was grouped by the cut-off point of 0.278 according to our prior study.

2 Supplementary Figures and Tables

2.1 Supplementary Tables

Supplementary Table 1. Subgroups and Normal range of laboratory variables in our hospital.

Variables	Group 1	Group 2	Normal range
HCT	Normal	Abnormal	0.4-0.5
WBC (10^9/L)	Normal	Abnormal	3.5-9.5
PLT (10^9/L)	Normal	Abnormal	100-350
hsCRP (mg/L)	0-3	> 3	0-3
RDW	≤ 0.15	> 0.15	0.115-0.15
BUN (mmol/L)	≤ 8.2	> 8.2	2.4-8.2
Cr (μmol/L)	≤ 116	> 116	31.8-116
ALB (g/L)	< 36	≥ 36	36-51
percentage of neutrophils			0.4-0.75
percentage of lymphocytes			0.2-0.4
NLR	≤ 3.75	> 3.75	percentage of neutrophils/percentage of lymphocytes 1-3.75

Supplementary Table 2. Putative risk factors for sepsis

Characteristic	Model 4	
	Adjusted RR[a]	95%CI
**Timing of surgery**		
elective	Ref.	
emergency	6.60	(5.08, 8.57)
**Type of surgery**		
abdominal and urogenital surgery	Ref.	
cardiovascular and thoracic surgery	1.35	(0.95, 1.92)
neurosurgery	3.84	(2.73, 5.41)
orthopedic surgery	0.47	(0.31, 0.72)
transplantation	4.17	(2.37, 7.34)
head and neck surgery	0.16	(0.07, 0.36)
**Duration of surgery (min)**		
≤ 120	Ref.	
> 120	2.88	(2.24, 3.71)
**Administration of dexmedetomidine**		
No	Ref.	
Yes	1.07	(0.87, 1.32)
**Administration of ulinastatin**		
No	Ref.	
Yes	2.81	(2.25, 3.51)
**Administration of steroids**		
No	Ref.	
Yes	2.21	(1.79, 2.72)
**WBC**		
Normal[b]	Ref.	
Abnormal[c]	1.86	(1.47, 2.35)
**HCT**		
Normal[b]	Ref.	
Abnormal[c]	1.51	(1.18, 1.93)
**RDW**		
≤ 0.15	Ref.	
> 0.15	1.53	(1.17, 2.00)
**PLT**		
Normal[b]	Ref.	
Abnormal[c]	1.33	(0.99, 1.78)
**ALB (g/L)**		
≥ 36	Ref.	
< 36	2.28	(1.83, 2.83)
**BUN (mmol/L)**		
≤ 8.2	Ref.	
> 8.2	2.11	(1.65, 2.71)
**SCr**		
≤ 116	Ref.	
> 116	2.57	(1.97, 3.36)
**GLU (mmol/L)**		
≤ 10	Ref.	
> 10	1.93	(1.40, 2.66)
**hsCRP (mg/L)**		
≤ 3	Ref.	
> 3	0.99	(0.53, 1.85)
**CAR**		
< 0.278	Ref.	
≥ 0.278	2.39	(1.88, 3.05)
**NLR**		
≤ 3.75	Ref.	
> 3.75	2.09	(1.69, 2.59)

[a]The relative risk is estimated from the method of Mantel-Haenszel based on the tree model 4 including all the potential confounding factors which are unimputed.[b] Normal WBC, HCT, and PLT is referred to 3.5 - 9.5(109/L), 0.4 - 0.5; 100 - 350(109/L),respectively.[c] Abormal WBC, HCT, PLT are referred to the values beyond the normal range respectively.

2.2 Supplementary Figures

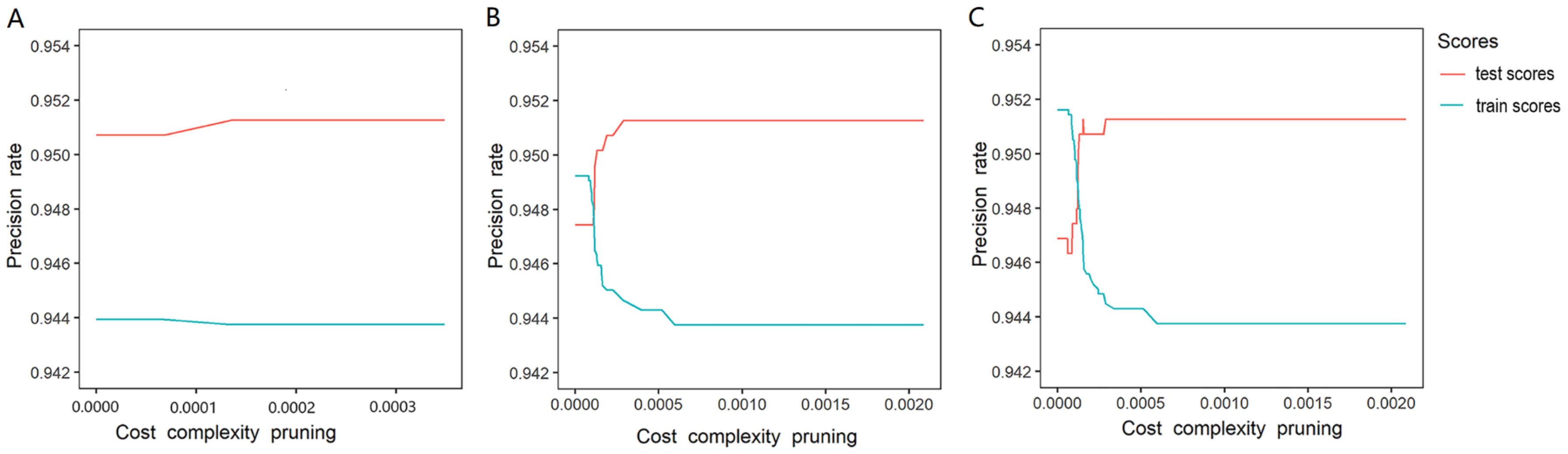

Supplementary Figure 1. Cost complexity pruning for details of tree models. **(A)**Pruning of Model 1 including only demographic factors. **(B)** Pruning of Model 2 including demographic factors and preoperative comorbidities. **(C)** Pruning of Model 3 including all the potential confounding factors.

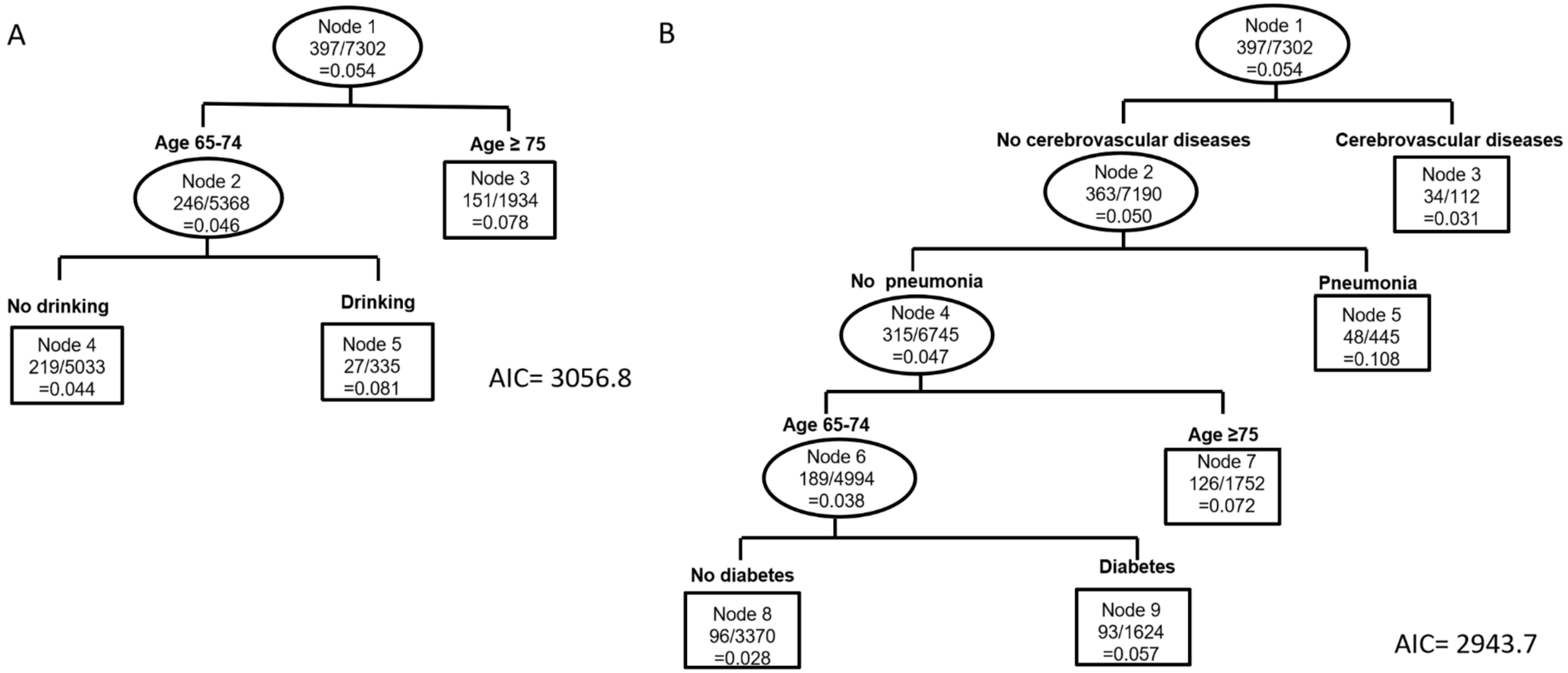

Supplementary Figure 2. Other tree models derived by different confounding factors. **(A)**Tree structure derived from only demographic factors (Model 1). **(B)**Tree structure derived from demographic factors and preoperative comorbidities (Model 2).

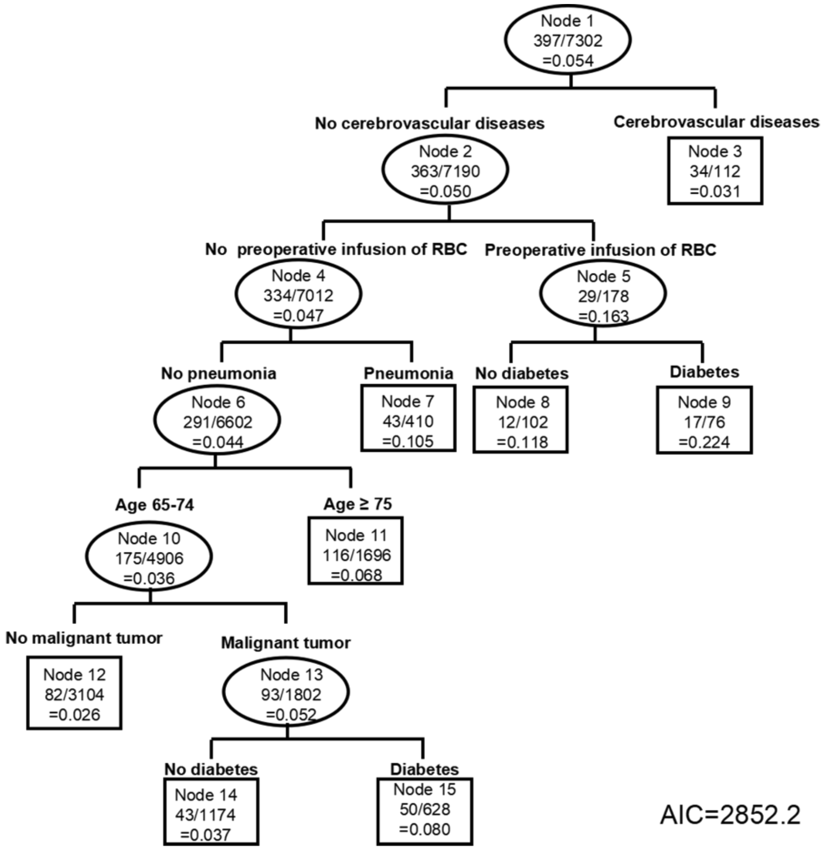

Supplementary Figure 3. Tree structure derived from all the confounding factors before data imputation (Model 4)
